# Supplementary material for: Quantitative evaluation of large corporate climate action initiatives shows mixed progress in their first half-decade
Source: Nat Commun. 2023 Jun 13;14:3487. doi: 10.1038/s41467-023-38989-2 (PMC10264448; doi:10.1038/s41467-023-38989-2)
Supplement: Supplementary file 1 — Supplementary Information [file 41467_2023_38989_MOESM1_ESM.pdf]

Supplementary Material: Quantitative evaluation of large corporate climate action initiatives shows mixed progress in their first half-decade

Ivan Ruiz Manuel *[1] and Kornelis Blok[2]

[1]Institute for Environmental Sciences, University of Geneva, Geneva, Switzerland.
[2]Faculty of Technology, Policy and Management, Delft University of Technology, Delft, Netherlands

May 18, 2023

Summary of reasons for omitting participating companies

Due to very low transparency or lack of quality data, some companies could not be effectively evaluated. While we included them in the "Characterising participation" section, they were excluded from further analysis, resulting in a total of nine companies being excluded.

Here we list some of the issues encountered during the data collection phase:

- Cases where companies had less than three submitted responses to CDP, or their CDP responses were private and not accessible to the public, and they did not report any environmental data in their annual reports.
- Cases where companies only reported emissions data in their annual reports as percentage reductions, making it impossible to accurately quantify their emissions.
- Cases where companies had longitudinal inconsistencies in the energy data reported in annual reports that could not be explained away by using other documents.
- Cases where companies released reports or submitted CDP questionnaires with a two-year delay, meaning 2019 values were not available at the time of data collection.
- Cases where companies were recent spin-offs with no existing documentation on the share of emissions attributed to the division before it was spun out.

*ivanruizmanuel@gmail.com

Environmental data collection

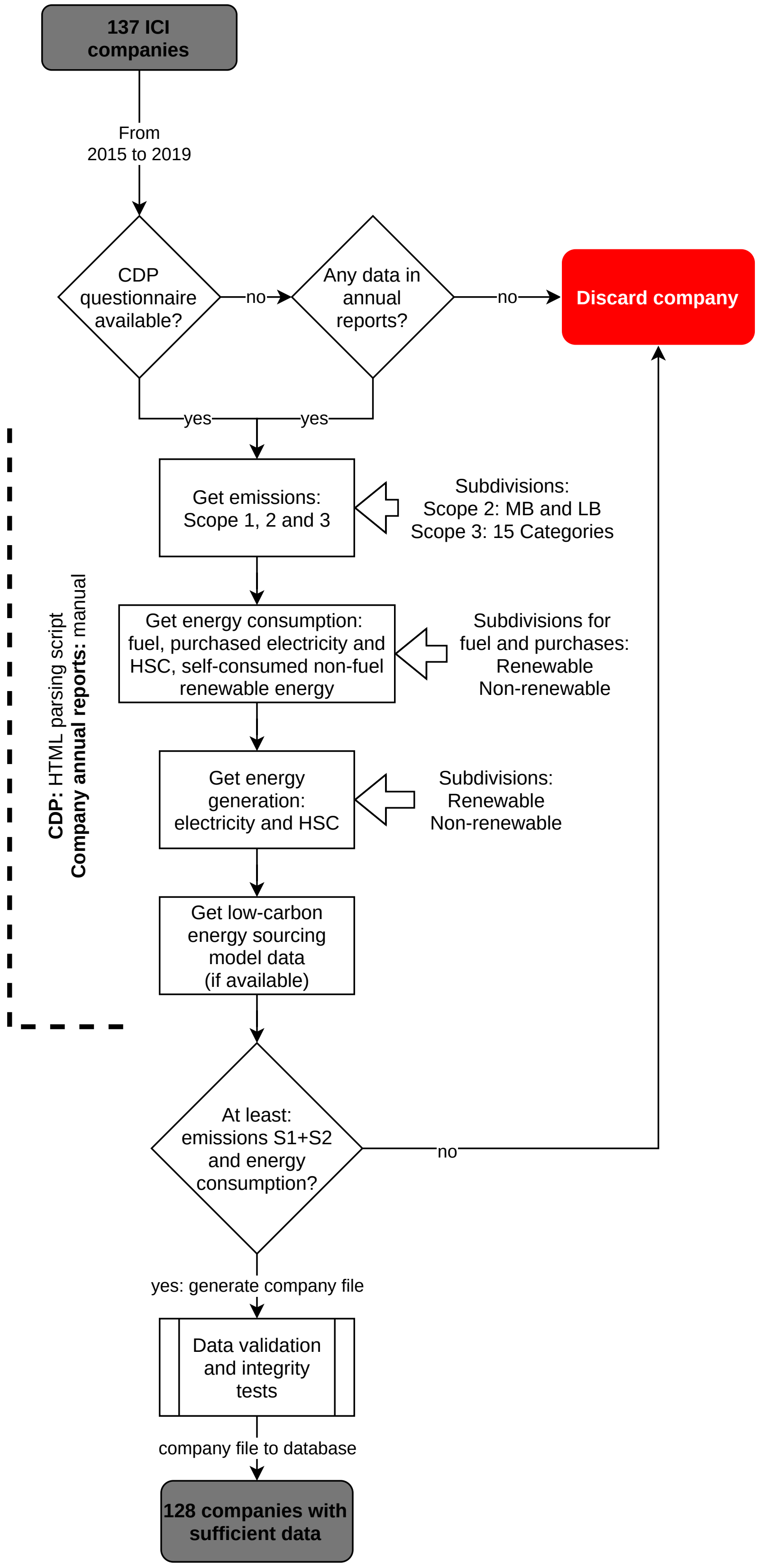

Supplementary Figure 1: **Methodology followed to gather environmental data for all SBTi and RE100 participants in the G500.**

Re-categorisation of the low-carbon energy sourcing model section in CDP questionnaires

Supplementary Table 1: Classifications used for low-carbon energy purchases in the study and as seen in different versions of the CDP questionnaire. Self owned and Grid mix categories were removed once identified as they violate GHG protocol requirements for Scope 2 Market-based reporting. "Check" was used as a trigger for manual validation, and covers all cases where a company did not follow the categories of that specific version of the questionnaire.

Study	CDP 2020	CDP 2019-2018	CDP 2017-2016
PPA direct line	Power purchase agreement (PPA) with on-site/off-site generator owned by a third party with no grid transfers (direct line)	Off-grid energy consumption from an on-site installation or through a direct line to an off-site generator owned by another company	Off-grid energy consumption from an on-site installation or through a direct line to an off-site generator owned by another company
PPA w/EAC	Power purchase agreement (PPA) with a grid-connected generator with energy attribute certificates	Power Purchase Agreement (PPA) with energy attribute certificates	Direct procurement contract with a grid-connected generator or Power Purchase Agreement (PPA), supported by energy attribute certificates
PPA no EAC	Power purchase agreement (PPA) with a grid-connected generator without energy attribute certificates	Power Purchase Agreement (PPA) without energy attribute certificates	Direct procurement contract with a grid-connected generator or Power Purchase Agreement (PPA), where electricity attribute certificates do not exist or are not required for a usage claim
Green Utility Product w/EAC	Green electricity products (e.g. green tariffs) from an energy supplier, supported by energy attribute certificates	Contract with suppliers or utilities (e.g. green tariff), supported by energy attribute certificates	Contract with suppliers or utilities, supported by energy attribute certificates
Green Utility Product no EAC	Green electricity products (e.g. green tariffs) from an energy supplier, not supported by energy attribute certificates	Contract with suppliers or utilities (e.g. green tariff), not supported by electricity attribute certificates	Contract with suppliers or utilities, with a supplier-specific emission rate, not backed by electricity attribute certificates
Unbundled EAC	Unbundled energy attribute certificates, Guarantees of Origin	Energy attribute certificates, Guarantees of Origin	Energy attribute certificates, Guarantees of Origin
	Unbundled energy attribute certificates, Renewable Energy Certificates (RECs)	Energy attribute certificates, Renewable Energy Certificates (RECs)	Energy attribute certificates, Renewable Energy Certificates (RECs)
	Unbundled energy attribute certificates, International REC Standard (I-RECs)	Energy attribute certificates, I-RECs	Energy attribute certificates, I-RECs
	Unbundled energy attribute certificates, other - please specify	-	-
Grid mix	-	Grid mix of renewable electricity	-
Self owned	-	-	Grid-connected electricity generation owned, operated or hosted by the company, where electricity attribute certificates do not exist or are not required for a usage claim
	-	-	Grid-connected generation owned, operated or hosted by the company, with energy attribute certificates created and retired by company
Check	Other, please specify	Other, please specify	Other (specify in Comment column)
	-	-	Off-grid energy consumption from an onsite installation or through a direct line to an off-site generator

Energy sector categorisation

Fortune's[1] list contains both sector and industry categories, broadly based on the GICS[2] classification of the company. These were re-classified into six energy categories. Companies labelled as "Energy" by Fortune were reclassified into other existing categories by consulting other business websites such as Fidelity, Forbes or Bloomberg, or by reading company reports. "Mining, Crude-Oil Production" was subdivided into "Mining (Metals)", "Mining (Coal)" and "Crude-Oil Production" by following a similar method. Mining companies with some fossil extraction but otherwise very diversified portfolios were set as "Mining (Metals)". The final list of energy use sectors has the following six categories:

- Electricity Generation: electric and multi-utilities with significant electricity production.
- Fossil Fuel Production: oil and gas producers, and coal companies.
- Energy Intensive Industry: metal, cement, glass and paper producer.
- Light Industry: any industrial that does not classify as energy intensive.
- Transport: includes businesses with a focus on transportation by railway, sea and air (car manufacturers are not included).
- Services: companies whose activities do not entail manufacturing or extraction of physical goods. Financial, software companies and retail fall into this category.

Supplementary Table 2: Summary of energy sector re-classification of the industry classification in the Fortune G500 2020 list. Fortune G500 industries are subdivided by semicolons.

**Energy sector**	**Fortune G500 Industries**
Electricity Generation	Utilities
Energy Intensive Industry	Metals; Chemicals; Building Materials, Glass
Fossil Fuel Production	MotorPetroleum Refining; Pipelines;<br>Oil & Gas; Crude-Oil Production; Mining (Coal)
Light Industry	Motor Vehicles & Parts; Aerospace & Defense; Apparel;<br>Engineering & Construction; Pharmaceuticals; Textiles;<br>Electronics, Electrical Equip.; Food Production; Semiconductors;<br>Industrial Machinery; Motor Vehicles; Motor Vehicle Parts;<br>Real estate; Computers; Electronics; Computers, Office Equipment;<br>Consumer Food Products; Construction and Farm Machinery;<br>Network and Other Communications Equipment; Mining (Metals);<br>Soaps and Cosmetics; Medical Products and Equipment; Tobacco;<br>Home Equipment, Furnishings; Oil & Gas Equipment, Services;<br>Technology Hardware, Storage and Peripherals; Beverages;<br>Semiconductors and Other Electronic Components
Services	Banks: Commercial and Savings; Insurance: Life, Health;<br>Food & Drug Stores; Trading; Telecommunications;<br>Specialty Retailers; Diversified Financials; Megabanks;<br>Insurance: Property and Casualty; Food Services;<br>Insurance: Life and Health; Wholesalers: Health Care;<br>Internet Services and Retailing; Entertainment;<br>Health Care: Insurance and Managed Care; Computer Software;<br>Information Technology Services; Diversified Outsourcing Services;<br>Wholesalers: Food and Grocery; General Merchandisers;<br>Health Care: Medical Facilities; Superregional Banks;<br>Consumer Credit Card and Related Services;<br>Wholesalers: Electronics and Office Equipment;<br>Health Care: Pharmacy and Other Services;<br>Interactive Media and Services
Transport	Airlines; Delivery; Railroads; Mail, Package, and Freight Delivery;<br>Shipping; Trucking, Transportation, Logistics

Summary of data issues and methodological errors in CDP and company reports

Once data was obtained, it was subjected to a series of validation tests to ensure its quality due to two issues: yearly errors and longitudinal errors (i.e. those affecting several consecutive years). Yearly errors were mostly caused by the fact that CDP does not subject their own questionnaires to any examination or edits after submission[3], leaving the possibility of human error open. Reasons for longitudinal errors are harder to assess: they went from simple confusion on how the GHG Protocol should be applied to outright resistance to new guidelines in CDP questionnaires.

The following list addresses some of the most common issues seen in both CDP and annual report data:

- Yearly errors (individual submission):
  - Magnitude errors: e.g. submitting energy in kWh instead of the requested MWh to CDP, or a company's own annual reports.
  - Conversion errors: e.g. not converting energy from TJ to MWh or vice-versa.
  - Typing errors: e.g. adding or omitting digits in energy use or emissions values, altering by several orders of magnitude.
  - Category errors: e.g. flipping renewable and non-renewable energy values, or location-based and market-based scope 2 emissions.
  - Empty categories: e.g. disclosing renewable fuel and non-renewable fuel, but leaving the total fuel value empty.
  - Equality errors: e.g. total energy not being equal to the sum of total renewable energy and total non-renewable energy in the CDP questionnaire.
- Longitudinal errors (year-by-year):
  - Inconsistent accounting boundaries: e.g. arbitrarily removing and re-including subsidiaries within the reporting boundary without clear changes in the company's operations.
  - Pervasive methodology oversights: e.g. conversion errors that remained uncorrected for several years or misunderstandings in GHG Protocol guidelines.
  - Inconsistent ownership of on-site generation and direct line consumption: e.g. alternating the reporting solar panels as self-owned or a direct line PPA throughout the years.
  - Resistance to CDP updates: companies often opted to submit information using older CDP guidelines, leading to invalid data and even GHG Protocol violations. E.g. submitting renewable energy in the grid-mix as a valid Scope 2 MB purchase even through the protocol's guidelines explicitly state that this should not be done[4].

Testing methods had to be developed to remove or minimise the effect of these oversights for each company, which are described in the methodology and other sections of this supplementary material.

Final cross-year consistency test

Supplementary Fig. 2 depicts the year-by-year evolution of the emission factors (Equation 11 to Equation 13) of each company after normalising them using the earliest available year. For a limited number of cases, a correction could not be applied or was not merited. For **Scope 1**: Hewlett Packard Enterprise went through heavy restructuring throughout the early years of this study, so its disclosed data was kept as-is. For **Scope 2 MB/LB**: Both companies exceeding the 90% deviation limit (Apple, Swiss RE) had a high share of renewable purchased energy ($> 90\%$) and no indication of errors in their accounting, so their values were kept as they were.

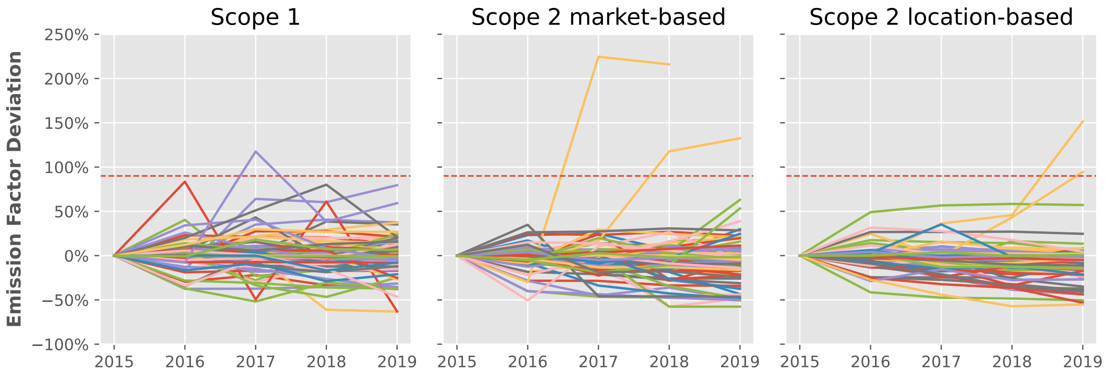

Supplementary Figure 2: **Evolution of the emission factors of all companies with targets, excluding utilities** ($n = 97$)**.** The red line represents the limit at which a company was reviewed.

Comparison of Ambition indicators against global SSP scenarios

For completeness, here we present ambition against the global scenarios. Fig. 2 and Fig. 3 in the study assume companies operate primarily within the OECD. We opted to present OECD trends since these companies are mostly headquartered within this region, meaning a fair amount of their operations are likely still concentrated within it.

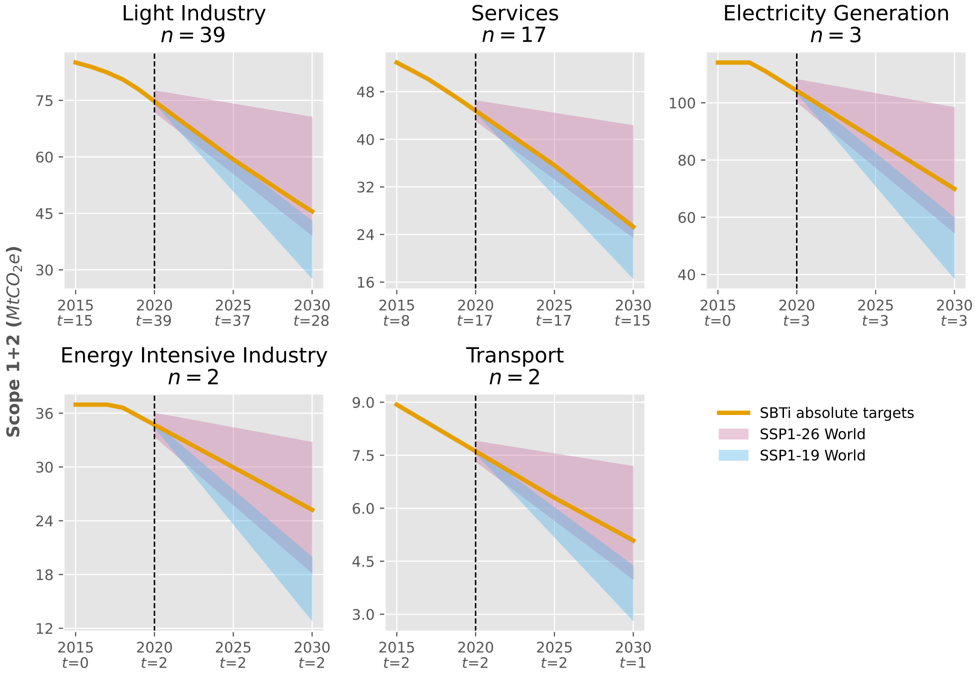

Supplementary Figure 3: **Ambition of companies with absolute targets in the Science-Based Targets initiative (SBTi) compared to global scenarios.** Grouped by sector. Targets were compared against global scenarios under Shared Socioeconomic Pathways with low challenges to mitigation and adaptation, keeping global warming below 1.5°C (SSP1-19) or 2°C (SSP1-26). t represents the number of companies with active targets in a year.

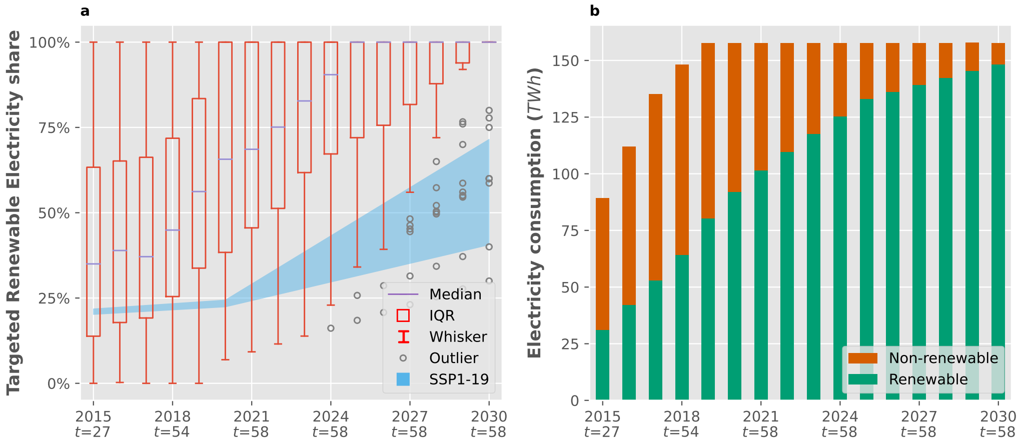

Supplementary Figure 4: **Ambition of RE100 members ($n = 58$) compared to global scenarios**. Members without approved targets were excluded in the initial years. t represents the number of companies with active targets each year. **a** Box plot distribution of the renewable electricity ratio targeted by each company compared against global SSP scenarios of the ratio of renewable electricity. Boxes envelop the interquartile range (IQR) of the data with the median as a line (25th, 50th and 75th percentiles). Whiskers stretch from the box by 1.5x of the IQR. Values outside this range are shown as outliers. **b** Growth of renewable electricity consumption in the initiative if targets are met, assuming total consumption remains constant after 2019.

Energy consumption statistics

Supplementary Table 3: Statistics of a linear regression of the sums of all companies for each energy type (see Methodology) against the five years where information was collected. Energy sector and sample group are presented in *italics*. AAGR represents the average annual growth rate.

				Sum						
**Sector**	**Group**	**Companies**	**Type**	**2015 (TWh)**	**2019 (TWh)**	**AAGR**	**slope**	r	r^2	**SE**
			RE Self-Gen	0.618	0.017	-33.15%	-0.17	-0.78	0.608	0.079
			RE Fuel	10.709	10.686	0.35%	0.103	0.294	0.087	0.194
Energy Intensive Industry	*SBTi only*	3	NRE Fuel	309.611	251.833	-5.02%	-14.086	-0.993	0.985	1
			RE Purchases	0.121	5.389	707.64%	1.191	0.912	0.832	0.309
			NRE Purchases	35.254	23.616	-9.33%	-2.551	-0.94	0.883	0.537
			RE Self-Gen	0.385	0.53	9.91%	0.041	0.841	0.708	0.015
			RE Fuel	1.992	2.472	6.91%	0.176	0.742	0.55	0.092
	SBTi only	26	NRE Fuel	97.235	96.573	-0.04%	0.232	0.089	0.008	1.502
			RE Purchases	7.626	19.029	26.10%	2.718	0.99	0.98	0.222
			NRE Purchases	71.211	61.392	-3.43%	-2.647	-0.87	0.757	0.866
			RE Self-Gen	0.181	1.525	71.83%	0.314	0.948	0.898	0.061
			RE Fuel	0.7	2.197	58.21%	0.251	0.62	0.384	0.184
Light Industry	*RE100 only*	5	NRE Fuel	17.786	16.442	-1.90%	-0.337	-0.878	0.77	0.106
			RE Purchases	2.108	5.669	28.87%	0.917	0.991	0.983	0.07
			NRE Purchases	16.148	9.727	-11.26%	-1.612	-0.95	0.902	0.307
			RE Self-Gen	0.057	0.126	23.97%	0.015	0.855	0.731	0.005
			RE Fuel	4.95	5.138	3.28%	-0.011	-0.03	0.001	0.212
	Overlap	14	NRE Fuel	55.392	54.562	-0.09%	0.304	0.16	0.026	1.086
			RE Purchases	3.319	11.635	38.19%	2.254	0.978	0.956	0.278
			NRE Purchases	33.857	26.412	-5.91%	-1.819	-0.926	0.857	0.43
			RE Self-Gen	0.001	0.06	458.58%	0.016	0.99	0.98	0.001
			RE Fuel	0.174	1.068	134.83%	0.268	0.863	0.745	0.09
	SBTi only	9	NRE Fuel	9.062	8.242	-1.65%	-0.319	-0.545	0.297	0.283
			RE Purchases	0.74	3.976	57.55%	0.707	0.876	0.767	0.225
			NRE Purchases	32.807	26.67	-4.94%	-1.472	-0.904	0.817	0.403
			RE Self-Gen	0.025	0.03	8.35%	0.003	0.553	0.305	0.002
			RE Fuel	0.095	0.08	-4.12%	-0.005	-0.861	0.741	0.002
Services	*RE100 only*	27	NRE Fuel	4.333	3.946	-2.10%	-0.099	-0.677	0.458	0.062
			RE Purchases	6.928	26.473	41.18%	5.075	0.994	0.988	0.322
			NRE Purchases	24.627	12.427	-15.37%	-3.23	-0.968	0.937	0.483
			RE Self-Gen	0.487	0.154	-1.51%	-0.063	-0.58	0.336	0.051
			RE Fuel	0.071	0.381	428.77%	0.097	0.369	0.136	0.141
	Overlap	11	NRE Fuel	23.991	24.763	0.81%	0.261	0.842	0.709	0.096
			RE Purchases	10.98	27.978	26.74%	4.327	0.997	0.994	0.191
			NRE Purchases	60.944	44.864	-7.37%	-4.06	-0.999	0.998	0.11
			RE Self-Gen	0.001	0.03	2727.54%	0.002	0.138	0.019	0.008
			RE Fuel	0.267	0.176	-2.24%	-0.01	-0.316	0.1	0.017
	SBTi only	1	NRE Fuel	13.17	12.595	-1.10%	-0.156	-0.827	0.683	0.061
			RE Purchases	0.947	4.203	71.26%	0.735	0.869	0.755	0.242
Transport			NRE Purchases	10.003	5.972	-11.41%	-0.943	-0.92	0.846	0.232
			RE Self-Gen	0	0	NaN	0	-0.177	0.031	0
			RE Fuel	0	0	NaN	0	-0.018	0	0
	Overlap	1	NRE Fuel	2.248	1.63	-7.44%	-0.157	-0.89	0.793	0.046
			RE Purchases	0.01	0.6	708.02%	0.154	0.912	0.832	0.04
			NRE Purchases	0.732	0.113	-32.61%	-0.155	-0.904	0.816	0.042
			RE Self-Gen	1.755	2.47	11.08%	0.157	0.744	0.554	0.081
			RE Fuel	18.958	22.198	4.60%	0.87	0.783	0.613	0.4
			NRE Fuel	532.829	470.586	-3.04%	-14.356	-0.975	0.95	1.899
			RE Purchases	32.778	104.951	34.43%	18.078	1	0.999	0.299
Totals	*All*	97	NRE Purchases	285.583	211.194	-7.24%	-18.489	-0.995	0.989	1.106
			Total RE	53.491	129.62	25.01%	19.106	0.998	0.996	0.707
			Total NRE	818.412	681.78	-4.45%	-32.845	-0.99	0.98	2.725
			Total Fuel	551.787	492.784	-2.78%	-13.486	-0.975	0.951	1.76
			Total Purchases	318.361	316.145	-0.16%	-0.411	-0.254	0.065	0.903
			Total Energy	871.903	811.4	-1.77%	-13.739	-0.959	0.921	2.329

GHG protocol scope statistics

Supplementary Table 4: Statistics of a linear regression of the sums of all companies for Scope 1, Scope 2 and Scope 1+2 against the five years where information was collected. Scope 2 MB data preferred when available. Energy sector and sample group are presented in *italics*. AAGR represents the average annual growth rate.

				**Sum**						
**Sector**	**Group**	**Companies**	**Type**	**2015 (TWh)**	**2019 (TWh)**	**AAGR**	**slope**	**r**	**r^2**	**SE**
			S1	371.639	196.736	-14.55%	-43.023	-0.994	0.987	2.833
Electricity Generation	*SBTi only*	5	S2	19.399	12.510	-10.299%	-1.811	-0.979	0.959	0.215
			S1+2	391.038	209.246	-14.345%	-44.835	-0.995	0.990	2.622
			S1	206.594	150.672	-7.55%	-14.105	-0.991	0.981	1.119
Energy Intensive Industry	*SBTi only*	3	S2	20.807	11.533	-13.39%	-2.111	-0.934	0.872	0.466
			S1+2	227.401	162.205	-8.07%	-16.216	-0.992	0.983	1.222
			S1	27.757	26.297	-1.17%	-0.306	-0.482	0.232	0.321
	SBTi only	26	S2	35.826	25.576	-7.78%	-2.775	-0.95	0.903	0.524
			S1+2	63.583	51.872	-4.74%	-3.082	-0.913	0.833	0.796
			S1	6.453	6.393	0.17%	0.037	0.159	0.025	0.132
Light Industry	*RE100 only*	*5*	S2	8.672	6.473	-6.62%	-0.664	-0.908	0.825	0.177
			S1+2	15.126	12.866	-3.77%	-0.628	-0.891	0.794	0.185
			S1	12.704	12.144	-1.02%	-0.069	-0.329	0.108	0.115
	Overlap	14	S2	16.363	11.867	-7.63%	-1.114	-0.972	0.944	0.156
			S1+2	29.067	24.011	-4.61%	-1.183	-0.95	0.902	0.226
			S1	5.191	3.969	-6.40%	-0.281	-0.96	0.922	0.047
	SBTi only	9	S2	16.19	11.572	-7.85%	-1.096	-0.958	0.918	0.189
			S1+2	21.38	15.541	-7.53%	-1.377	-0.966	0.932	0.214
			S1	1.611	1.413	-3.13%	-0.055	-0.886	0.786	0.016
Services	*RE100 only*	27	S2	11.691	5.391	-17.37%	-1.652	-0.977	0.955	0.207
			S1+2	13.302	6.805	-15.23%	-1.706	-0.976	0.953	0.22
			S1	9.592	9.756	0.50%	-0.026	-0.156	0.024	0.094
	Overlap	11	S2	28.406	19.98	-8.39%	-2.134	-0.994	0.989	0.132
			S1+2	37.999	29.736	-5.93%	-2.159	-0.993	0.987	0.143
			S1	3.772	3.778	0.06%	-0.002	-0.051	0.003	0.021
	SBTi only	1	S2	5.508	4.025	-7.36%	-0.384	-0.96	0.922	0.064
Transport			S1+2	9.281	7.803	-4.17%	-0.386	-0.942	0.887	0.08
			S1	0.402	0.37	-2.03%	-0.009	-0.946	0.896	0.002
	Overlap	1	S2	0.095	0.004	-37.12%	-0.024	-0.9	0.81	0.007
			S1+2	0.497	0.375	-6.62%	-0.033	-0.924	0.853	0.008
			S1	645.716	411.529	-10.61%	-57.839	-0.997	0.995	2.451
Totals	*All*	102	S2	162.957	108.931	-9.544%	-13.765	-0.994	0.987	0.898
			S1+2	808.673	520.459	-10.403%	-71.604	-0.999	0.998	2.062

Supplementary Table 5: **Disaggregation of substantive progress by GHG Scope, in $MtCO_2e$.** The data featured in Fig. 11d is separated by sector and GHG Protocol scope, including Location Based (LB) and Market Based (MB) approaches for Scope 2[4]. Due to inconsistencies in emissions reporting, MB data was preferred if available. Otherwise, LB data was used.

Sector	Scope	2015	2016	2017	2018	2019
Electricity Generation	S1	371.64	330.9	302.39	250.47	196.74
	S2 LB	11.68	9.23	3.58	3.42	2.52
	S2 MB	7.72	8.41	11.11	9.89	9.99
Energy Intensive Industry	S1	206.59	194.98	173	165.77	150.67
	S2 LB	4.82	4.76	4.69	4.48	4.01
	S2 MB	15.99	10.67	9.52	8.39	7.53
Light Industry	S1	46.91	47.32	46.02	48.09	44.83
	S2 LB	15.44	13.88	11.04	7.06	7.42
	S2 MB	45.42	46.59	40.87	41.78	36.49
Services	S1	16.39	16.48	16.3	15.38	15.14
	S2 LB	26.68	7.7	6.07	5.84	5.38
	S2 MB	29.61	44.56	38.92	36.3	31.56
Transport	S1	4.17	4.26	4.29	4.2	4.15
	S2 LB	0.09	0.07	0	0	0
	S2 MB	5.51	5.14	5.12	4.28	4.03
Totals	S1	645.72	593.93	541.99	483.92	411.53
	S2 LB	58.71	35.65	25.38	20.8	19.33
	S2 MB	104.25	115.37	105.53	100.63	89.6

Target progress statistics

Contrary to previous studies, our analysis has a special focus on the effects of collective action in the initiatives. Regardless, the individual performance of members against their targets remains an important aspect of non-state action studies. The following table discloses member progress against the extended linearised targets seen in Fig. 7, Fig. 8 and Fig. 11.

Supplementary Table 6: **Target progress per initiative.** In the case of the SBTi progress has been broken up by scope, including Scope 2 category.

Initiative	Companies	N. on track	% on track	Target unit	Total 2019	T. on track 2019	% on track
SBTi abs. S1	63	46	73%	$MtCO2_e$	157.58	134.75	86%
SBTi abs. S2 LB	16	10	63%	$MtCO2_e$	27.62	19.19	69%
SBTi abs. S2 MB	47	36	77%	$MtCO2_e$	56.12	33.68	60%
SBTi int. S1	7	-	-	$MtCO2_e$	246.14	-	-
SBTi int. S2 LB	1	-	-	$MtCO2_e$	2.52	-	-
SBTi int. S2 MB	6	-	-	$MtCO2_e$	11.41	-	-
RE100	58	24	41%	TWh	73.69	30.30	41%

Wording in third-party verification assurance statements

In the study we group third-party verification into None, Limited, Moderate, Reasonable and High to reflect how it is classified in CDP questionnaires[5]. Comparison between verification methods is not straight since they vary depending on the type of company verified, the standard used, national law, etc. Regardless, a distinction can be made in the wording used by the auditor[6–8].:

- Negative wording: applies to Limited and Moderate assurance. In these cases the assurance statement will use negative wording such as "*nothing has come to our attention that causes us to believe that the non-financial statement is not in accordance with the applicable regulatory provisions*". Generally, this implies that the verification was limited to tasks such as inquiries and analytical procedures[6,9]. It does not imply that a company failed to obtain third-party verification, just that the level of scrutiny was not enough to achieve positive wording.

- Positive wording: applies to Reasonable and High assurance. The assurance statement will use positive wording such as "*in our opinion, the information were prepared, in all material aspects, in accordance with the guidelines*". It can be seen as a statement of opinion by the auditor and in general it implies higher levels of evaluation of the data and procedures of the company[9]. This level of assurance is usually more expensive[10].

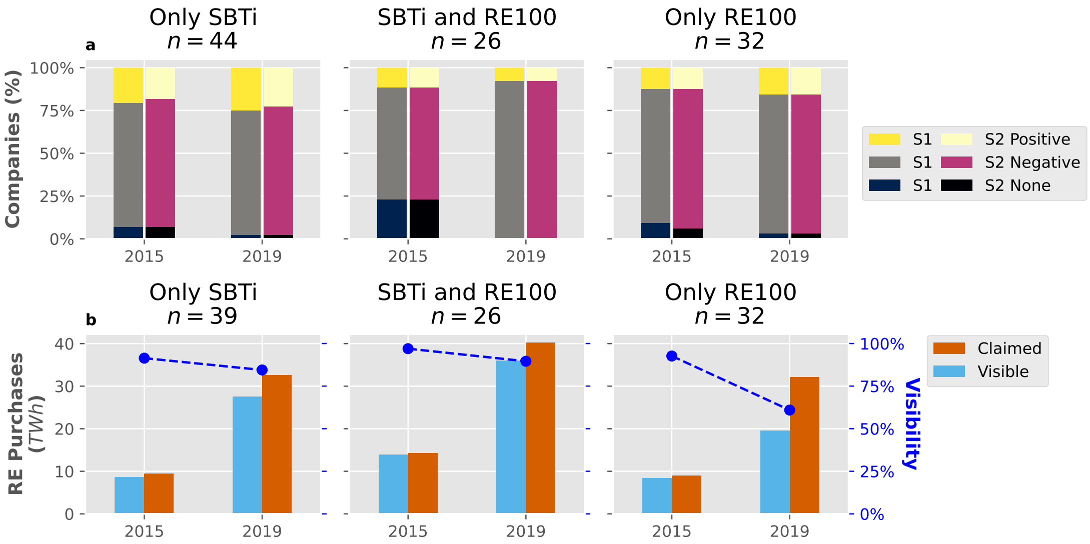

Supplementary Figure 5: **Robustness indicators for companies with targets set**. Separated into companies exclusively in the Science-Based Targets initiative (SBTi), those only in RE100 and companies enrolled in both. **a** Ratio of third-party verification according to the wording used in assurance statements for Scope 1 (S1) and Scope 2 (S2) emissions. **b** Comparison of claimed purchased renewable energy (RE) against purchases with a publicly visible sourcing model. Companies in the Electricity Generation sector were omitted.

Supplementary References

1. Fortune. *Global 500* https://fortune.com/global500/2020/. 2020.
2. S&P. *GICS: Global Industry Classification Standard* 2018.
3. CDP. *Terms and Conditions - CDP* https://www.cdp.net/en/info/terms-and-conditions (2022).
4. Sotos, M. *GHG Protocol Scope 2 Guidance: An Amendment to the GHG Protocol Corporate Standard* (ed World Resources Institute) ISBN: 978-1-56973-850-4 (World Resources Institute, 2015).
5. CDP. *Verification - CDP* https://www.cdp.net/en/guidance/verification. 2021.
6. IAASB. *ISAE 3000 (Revised), Assurance Engagements Other than Audits or Reviews of Historical Financial Information* Dec. 2013.
7. Institute of Chartered Accountants in England and Wales. *Elements of an Assurance Report* https://www.icaew.com/technical/audit-and-assurance/assurance/process/reporting/elements-of-an-assurance-report. 2021.
8. Fédération des Experts Comptables Européens. *Principles of Assurance: Fundamental Theoretical Issues with Respect to Assurance in Assurance Engagements* 2003.
9. American Institute of Certified Public Accountants. *AT Section 101 - Attest Engagements* 2016. https://us.aicpa.org/content/dam/aicpa/research/standards/auditattest/downloadabledocuments/at-00101.pdf (2023).
10. European Commission. *Summary Report of the Public Consultation on the Review of the Non-Financial Reporting Directive* June 11, 2020. https://eur-lex.europa.eu/legal-content/EN/TXT/PDF/?uri=PI_COM:Ares(2020)3997889&from=DE.
