# Supplementary material for: Quantitative evaluation of large corporate climate action initiatives shows mixed progress in their first half-decade
Source: Nat Commun. 2023 Jun 13;14:3487. doi: 10.1038/s41467-023-38989-2 (PMC10264448; doi:10.1038/s41467-023-38989-2)
Supplement: Supplementary file 3 — Reporting Summary [file 41467_2023_38989_MOESM3_ESM.pdf]

nature portfolio

Corresponding author(s): Ivan Ruiz Manuel
Last updated by author(s): April, 23, 2023

Reporting Summary

Nature Portfolio wishes to improve the reproducibility of the work that we publish. This form provides structure for consistency and transparency in reporting. For further information on Nature Portfolio policies, see our Editorial Policies and the Editorial Policy Checklist.

Statistics

For all statistical analyses, confirm that the following items are present in the figure legend, table legend, main text, or Methods section.

n/a	Confirmed	
☐	☒	The exact sample size (*n*) for each experimental group/condition, given as a discrete number and unit of measurement
☒	☐	A statement on whether measurements were taken from distinct samples or whether the same sample was measured repeatedly
☒	☐	The statistical test(s) used AND whether they are one- or two-sided<br>*Only common tests should be described solely by name; describe more complex techniques in the Methods section.*
☒	☐	A description of all covariates tested
☒	☐	A description of any assumptions or corrections, such as tests of normality and adjustment for multiple comparisons
☐	☒	A full description of the statistical parameters including central tendency (e.g. means) or other basic estimates (e.g. regression coefficient) AND variation (e.g. standard deviation) or associated estimates of uncertainty (e.g. confidence intervals)
☒	☐	For null hypothesis testing, the test statistic (e.g. *F*, *t*, *r*) with confidence intervals, effect sizes, degrees of freedom and *P* value noted<br>*Give P values as exact values whenever suitable.*
☒	☐	For Bayesian analysis, information on the choice of priors and Markov chain Monte Carlo settings
☒	☐	For hierarchical and complex designs, identification of the appropriate level for tests and full reporting of outcomes
☒	☐	Estimates of effect sizes (e.g. Cohen's *d*, Pearson's *r*), indicating how they were calculated

Our web collection on statistics for biologists contains articles on many of the points above.

Software and code

Policy information about availability of computer code

Data collection	All code is available here: https://doi.org/10.4121/16616965
Data analysis	All data analysis code is available here: https://doi.org/10.4121/16616965

For manuscripts utilizing custom algorithms or software that are central to the research but not yet described in published literature, software must be made available to editors and reviewers. We strongly encourage code deposition in a community repository (e.g. GitHub). See the Nature Portfolio guidelines for submitting code & software for further information.

Data

Policy information about availability of data

All manuscripts must include a data availability statement. This statement should provide the following information, where applicable:

- Accession codes, unique identifiers, or web links for publicly available datasets
- A description of any restrictions on data availability
- For clinical datasets or third party data, please ensure that the statement adheres to our policy

The evaluated company data generated in this study have been deposited in the 4TU.ResearchData repository under accession code 10.4121/16616965.
The raw CDP questionnaire data are available under restricted access due to limits set by CDP, access can be obtained by registering in the CDP website at https://www.cdp.net/.
The scenario data used in the study are available in the IAMC 1.5° Scenario Explorer at https://data.ene.iiasa.ac.at/iamc-1.5c-explorer/.

Human research participants

Policy information about studies involving human research participants and Sex and Gender in Research.

Reporting on sex and gender	N/a
Population characteristics	N/a
Recruitment	N/a
Ethics oversight	N/a

Note that full information on the approval of the study protocol must also be provided in the manuscript.

Field-specific reporting

Please select the one below that is the best fit for your research. If you are not sure, read the appropriate sections before making your selection.

☐ Life sciences ☐ Behavioural & social sciences ☒ Ecological, evolutionary & environmental sciences

For a reference copy of the document with all sections, see nature.com/documents/nr-reporting-summary-flat.pdf

Life sciences study design

All studies must disclose on these points even when the disclosure is negative.

Sample size	N/a
Data exclusions	N/a
Replication	N/a
Randomization	N/a
Blinding	N/a

Behavioural & social sciences study design

All studies must disclose on these points even when the disclosure is negative.

Study description	N/a
Research sample	N/a
Sampling strategy	N/a
Data collection	N/a
Timing	N/a
Data exclusions	N/a
Non-participation	N/a
Randomization	N/a

Ecological, evolutionary & environmental sciences study design

All studies must disclose on these points even when the disclosure is negative.

Study description	A quantitative, ex-post analysis of Fortune Global 500 companies in the SBTi and RE100 initiatives using a logical framework to

Study description	analyse climate target ambition, data disclosure robustness, implementation effectiveness and substantial mitigation impact.<br>Companies were grouped hierarchically under the following structure: Global 500 -> Initiative (SBTi, RE100, None) -> Energy Sector
Research sample	List of Fortune Global 500 companies for 2020 (total of 500 companies, selected because it is ranked by revenue, which would ensure materiality in the evaluation).<br>List of SBTi members (total of 1205 companies, selected due to high mitigation estimates in ex-ante studies)<br>List of RE100 members (total of 289 companies, selected due to high mitigation estimates in ex-ante studies).
Sampling strategy	We used string-matching techniques (python library fuzzywuzzy) to identify overlaps between the G500 sample, the SBTi sample and the RE100 sample.<br>This yielded a total of 137 overlapping companies which was our final sample for the analysis.
Data collection	Data scrapping techniques, direct download of initiative datasets and collection of public CDP questionnaires and company reports.<br>Python scripts were used to extract company data from CDP HTML files. This was deposited in Excel sheets with validation tests coded into them. Each company's Excel file was evaluated for disclosure mistakes, using data from Public Reports to correct them when possible.<br>After all companies were validated, the resulting environmental data was collected in a dataset using the python library Pandas.
Timing and spatial scale	The G500 and all initiative members were taken on February 2021. Members were not updated afterwards.<br>The study spans from 2015 to 2019. Later years were not collected because CDP responses were not available for them.
Data exclusions	9 companies (either committed or with approved targets) were omitted from the study due to disclosure issues, and are not in the final dataset.<br>Environmental data was not collected for non-initiative companies.<br>For Electric Utilities, only emissions data was collected due to missing data and disclosure issues in CDP questionnaires.
Reproducibility	Our validation tests were designed to be in the form of formal verification. All initiative data collected is available at https://doi.org/10.4121/16616965<br>We repeated our tests for approximately ~33% of the final list of 137 companies, finding similar results (emissions were within 5% variation). However, reproducibility might be affected by the availability of public reports released by these companies. We did not collect these in our database. However, we listed the reports used in for each company in the Excel files and they should be available at each company's website.
Randomization	Randomization was only applied to evaluate 50% of our sample of 137 companies to remove subsidiaries or identify cases were the string matching algorithm returned a false positive. The design of the study did not require randomization in any other step.
Blinding	Our study did not involve interviews or groups of participants. Collection and evaluation of environmental data involved one person (I.R.M.), so blinding was not possible.

Did the study involve field work? ☐ Yes ☒ No

Field work, collection and transport

Field conditions	N/a
Location	N/a
Access & import/export	N/a
Disturbance	N/a

Reporting for specific materials, systems and methods

We require information from authors about some types of materials, experimental systems and methods used in many studies. Here, indicate whether each material, system or method listed is relevant to your study. If you are not sure if a list item applies to your research, read the appropriate section before selecting a response.

Materials & experimental systems

n/a	Involved in the study
☒	☐ Antibodies
☒	☐ Eukaryotic cell lines
☒	☐ Palaeontology and archaeology
☒	☐ Animals and other organisms
☒	☐ Clinical data
☒	☐ Dual use research of concern

Methods

n/a	Involved in the study
☒	☐ ChIP-seq
☒	☐ Flow cytometry
☒	☐ MRI-based neuroimaging

Antibodies

Antibodies used: N/a

Validation: N/a

Eukaryotic cell lines

Policy information about cell lines and Sex and Gender in Research

Cell line source(s): N/a

Authentication: N/a

Mycoplasma contamination: N/a

Commonly misidentified lines (See ICLAC register): N/a

Palaeontology and Archaeology

Specimen provenance: N/a

Specimen deposition: N/a

Dating methods: N/a

☐ Tick this box to confirm that the raw and calibrated dates are available in the paper or in Supplementary Information.

Ethics oversight: N/a

Note that full information on the approval of the study protocol must also be provided in the manuscript.

Animals and other research organisms

Policy information about studies involving animals; ARRIVE guidelines recommended for reporting animal research, and Sex and Gender in Research

Laboratory animals: N/a

Wild animals: N/a

Reporting on sex: N/a

Field-collected samples: N/a

Ethics oversight: N/a

Note that full information on the approval of the study protocol must also be provided in the manuscript.

Clinical data

Policy information about clinical studies

All manuscripts should comply with the ICMJE guidelines for publication of clinical research and a completed CONSORT checklist must be included with all submissions.

Clinical trial registration	N/a
Study protocol	N/a
Data collection	N/a
Outcomes	N/a

Dual use research of concern

Policy information about dual use research of concern

Hazards

Could the accidental, deliberate or reckless misuse of agents or technologies generated in the work, or the application of information presented in the manuscript, pose a threat to:

No	Yes	
☒	☐	Public health
☒	☐	National security
☒	☐	Crops and/or livestock
☒	☐	Ecosystems
☒	☐	Any other significant area

Experiments of concern

Does the work involve any of these experiments of concern:

No	Yes	
☒	☐	Demonstrate how to render a vaccine ineffective
☒	☐	Confer resistance to therapeutically useful antibiotics or antiviral agents
☒	☐	Enhance the virulence of a pathogen or render a nonpathogen virulent
☒	☐	Increase transmissibility of a pathogen
☒	☐	Alter the host range of a pathogen
☒	☐	Enable evasion of diagnostic/detection modalities
☒	☐	Enable the weaponization of a biological agent or toxin
☒	☐	Any other potentially harmful combination of experiments and agents

ChIP-seq

Data deposition

☐ Confirm that both raw and final processed data have been deposited in a public database such as GEO.

☐ Confirm that you have deposited or provided access to graph files (e.g. BED files) for the called peaks.

Data access links<br>*May remain private before publication.*	N/a
Files in database submission	N/a
Genome browser session<br>(e.g. UCSC)	N/a

Methodology

Replicates	N/a
Sequencing depth	N/a

Antibodies	N/a
Peak calling parameters	N/a
Data quality	N/a
Software	N/a

Flow Cytometry

Plots

Confirm that:

- [ ] The axis labels state the marker and fluorochrome used (e.g. CD4-FITC).
- [ ] The axis scales are clearly visible. Include numbers along axes only for bottom left plot of group (a 'group' is an analysis of identical markers).
- [ ] All plots are contour plots with outliers or pseudocolor plots.
- [ ] A numerical value for number of cells or percentage (with statistics) is provided.

Methodology

Sample preparation	N/a
Instrument	N/a
Software	N/a
Cell population abundance	N/a
Gating strategy	N/a

- [ ] Tick this box to confirm that a figure exemplifying the gating strategy is provided in the Supplementary Information.

Magnetic resonance imaging

Experimental design

Design type	N/a
Design specifications	N/a
Behavioral performance measures	N/a

Acquisition

Imaging type(s)	N/a
Field strength	N/a
Sequence & imaging parameters	N/a
Area of acquisition	N/a

Diusion MRI ☐ Used ☒ Not used

Preprocessing

Preprocessing software	N/a
Normalization	N/a
Normalization template	N/a
Noise and artifact removal	N/a
Volume censoring	N/a

Statistical modeling & inference

Model type and settings	N/a
Effect(s) tested	N/a

Specify type of analysis: ☐ Whole brain ☐ ROI-based ☐ Both

Statistic type for inference (See Eklund et al. 2016)	N/a
Correction	N/a

Models & analysis

n/a	Involved in the study
☒	☐ Functional and/or effective connectivity
☒	☐ Graph analysis
☒	☐ Multivariate modeling or predictive analysis

Functional and/or effective connectivity	N/a
Graph analysis	N/a
Multivariate modeling and predictive analysis	N/a
